# Landscape fragmentation of the Natura 2000 network and its surrounding areas

**DOI:** 10.1371/journal.pone.0258615

**Published:** 2021-10-21

**Authors:** Alexandra Lawrence, Fabian Friedrich, Carl Beierkuhnlein

**Affiliations:** 1 Department of Biogeography, University of Bayreuth, Bayreuth, Germany; 2 BayCEER, Bayreuth Center for Ecology and Environmental Research, Bayreuth, Germany; 3 GIB, Bayreuth Institute for Geography, Bayreuth, Germany; Instituto Nacional de Pesquisas da Amazonia, BRAZIL

## Abstract

Habitat loss from anthropogenic development has led to an unprecedented decline in global biodiversity. Protected areas (PAs) exist to counteract this degradation of ecosystems. In the European Union, the Natura 2000 (N2k) network is the basis for continent-wide conservation efforts. N2k is the world’s largest coordinated network of protected areas. However, threats to ecosystems do not stop at the borders of PAs. As measured by a landscape fragmentation metric, anthropogenic development can affect the interiors of PAs. To ensure the long-term viability of the N2k network of PAs, this paper attempts to quantify the degree to which N2k sites are insulated from development pressures. We use a comprehensive dataset of effective mesh density (*seff*) to measure aggregate fragmentation inside and within a 5 km buffer surrounding N2k sites. Our results show a strong correlation (R² = 0.78) between fragmentation (*seff*) within and around N2k sites. This result applies to all biogeographical regions in Europe. Only a narrow majority (58.5%) of N2k sites are less fragmented than their surroundings. Remote and mountainous regions in northern Europe, the Alps, parts of Spain, and parts of eastern Europe show the lowest levels of fragmentation. These regions tend to hold the largest N2k sites as measured by area. In contrast, central and western Europe show the highest fragmentation levels within and around N2k sites. 24.5% of all N2k sites are classified as highly to very-highly fragmented. N2k PA age since initial protection does not correlate with the difference in exterior and interior fragmentation of N2k PAs. These results indicate that PAs in Europe are not sheltered from anthropogenic pressures leading to fragmentation. Hence, we argue that there is a high potential for improving PA efficacy by taking pre-emptive action against encroaching anthropogenic fragmentation and by targeting scarce financial resources where fragmentation pressures can be mitigated through enforced construction bans inside PAs.

## Introduction

Habitat loss from anthropogenic development is the primary driver of species extinctions worldwide, resulting in a rapid decline in global biodiversity [1–4]. As cities grow and transportation infrastructure expands, ecosystems are degraded, biodiversity is lost, and critical ecological functions are impaired [5–7]. To some degree, landscapes are fragmented by natural barriers. However, unlike most natural barriers, rapid growth of man-made infrastructure subdivides habitats into artificially small and isolated patches. This development has been illustrated in the first figure of the seminal book *Theory of Island Biogeography* [8], one of the foundational texts of modern nature conservation and planning. Nevertheless, there is still a deficit in the scientific understanding of the effects of fragmentation on biodiversity. Large and unfragmented stretches of natural land are highly desirable for habitat conservation. This should not diminish the importance of smaller, already fragmented habitats which are likewise essential for biodiversity protection [9, 10]. Yet increasingly small and isolated habitat patches are often ill-suited to achieve certain conservation goals, such as providing opportunities for species movement as an adaptive strategy in response to climate change [11]. Therefore, to counteract the adverse effects of rapid infrastructure development, protected areas (PAs) have been designated to protect and conserve threatened species, habitats, and ecosystems [12–14].

In 1992 the European Union established a special protected area network, Natura 2000 (N2k), which covers 18% of the EU’s terrestrial and 9.5% of its marine area [15]. This PA network includes Special Protection Areas (SPAs) (Birds Directive) and Special Areas of Conservation (SACs) (Habitats Directive). Together, they are considered to be among the most substantive international strategies for nature protection [16]. This network functions as a vital tool for the EU that is supposed to ensure the long-term persistence of Europe’s most threatened species and habitats [17].

The N2k network also meets Aichi Target 11, according to which every UN member nation except the United States agreed to protect 17% of terrestrial surface area and 10% of coastal and marine areas by 2020 (Target 11 of Aichi Biodiversity Targets) [18]. As part of the European strategy for green infrastructure, the N2k network further aims to improve connectivity between protected areas [19]. Overall, the establishment of the N2k network has increased both PA coverage and interconnectivity of PAs in the European Union [20]. To maximize spatial coverage of protected areas, policy makers frequently establish PAs within remote and mountainous regions where economic development pressures are typically less pronounced [21]. However, this emphasis on spatial coverage does not in itself ensure habitat quality, regulation enforcement, or overall effectiveness of PAs in biodiversity protection [21, 22]. Notably, there currently exists no EU-wide regulation strictly preventing new infrastructure from being built inside N2k PAs. Prevention of new infrastructure within N2k PAs is thus left to regulation and enforcement at the local and national level [23].

Within the borders of PAs, ecosystems are threatened due to increasing human pressure [20, 24]. The quality of PA surroundings has a strong influence on ecosystems within PAs [25–27]. Genetic diversity, for example, is severely impacted when dispersal of species from PAs is hampered by surrounding fragmentation [28, 29]. Conversely, healthy ecosystems in the vicinity of a PA may reduce isolation and contribute to population size and species persistence within the PA [28, 30, 31]. Research has shown that anthropogenic disturbances have steadily increased within PAs in the last decades, and smaller PAs are especially at risk of losing their effectiveness in conserving biodiversity in the face of ongoing infrastructure development [9, 32, 33].

To this day, the relationship between the quality of the surrounding matrix and the quality of habitat within the boundaries of PAs is a neglected field of research. A few studies of tropical ecosystems have shown that pressures stemming from the surrounding matrices of PAs are evident within PAs [24, 34]. It is still unclear to what extent these findings can be transferred to extratropical regions. As the historical cradle of industrialization and transportation infrastructure, Europe has one of the world’s heaviest human footprints. High anthropogenic pressure faces ecosystems across the continent [33–35]. The current dearth of broad-scale modelling approaches to analyse anthropogenic pressures in and around European PAs severely limits our understanding of effective biodiversity conservation on a continental scale [36].

In this study, we investigate anthropogenic fragmentation inside and around N2k sites across the EU. We define fragmentation as a landscape-scale process that includes (a) reduction in total habitat area, (b) increase in the number of habitat patches, and (c) decrease in sizes of habitat patches. We do not consider the degree of patch isolation. Thus, we measure habitat loss and fragmentation as a unified phenomenon rather than measuring fragmentation per se [37]. This is an important distinction because habitat loss is known to be a primary threat to biodiversity while the effects of landscape configuration, such as fragmentation per se, are debated [10, 38–40]. The most common view among ecologists is that both habitat amount and fragmentation per se result in negative consequences for biodiversity [7, 38, 41]. Fahrig [42] challenges this view by proposing the habitat amount hypothesis (HAH). The HAH predicts that variation in species richness among sampling sites can be explained by the amount of habitat in the local landscape around the sites, while the spatial configuration of habitat (e.g., fragmentation per se) makes little difference. This interpretation of the HAH has both defenders and critics [40–44]. In a review of 118 studies reporting significant ecological responses to fragmentation per se, Fahrig [45] shows that 70% of ecological responses to fragmentation per se are non-significant. Among the 381 significant ecological responses, 76% showed positive effects towards fragmentation per se, such as increased species abundance and richness. However, this study has been challenged for reliance on a small sample size of species and landscapes under study. Likewise, the study’s overall implications for conservation are controversial because they potentially lead to a skewed concept of neutral or positive effects of fragmentation per se on biodiversity [38, 44]. Unlike the contention surrounding the effects of fragmentation per se, habitat loss and fragmentation as one unified phenomenon, as measured in this study, is widely accepted as a major threat to biodiversity [41, 44].

Preliminary analysis by the EEA [46], investigating habitat loss and fragmentation as one unified phenomenon, suggest that N2k sites are, in general, less fragmented relative to their surroundings, and fragmentation varies among biogeographical regions. However, the EEA did not publish any quantitative data in support of this conclusion. Thus, conservation strategies based on these findings are missing critical information needed to address anthropogenic pressures in and around PAs.

This study seeks to investigate the relationship between fragmentation around N2k sites and fragmentation within N2k sites. We hypothesized that A) N2k sites are less fragmented than their surroundings; B) the least fragmented sites are located in remote and mountainous regions; and C) the degree of fragmentation within N2k sites correlates positively with the degree of fragmentation in the sites’ surroundings for all biogeographical regions. Further, we expected protected status to curb additional fragmentation within PAs while development continues relatively unabated in surrounding areas. Therefore, we hypothesized that (D) the difference between exterior and interior fragmentation of N2k sites has increased with time since N2ksites first gained protected status, from here on referred to as “age“.

## Methods

### Study area

**Fig 1:**
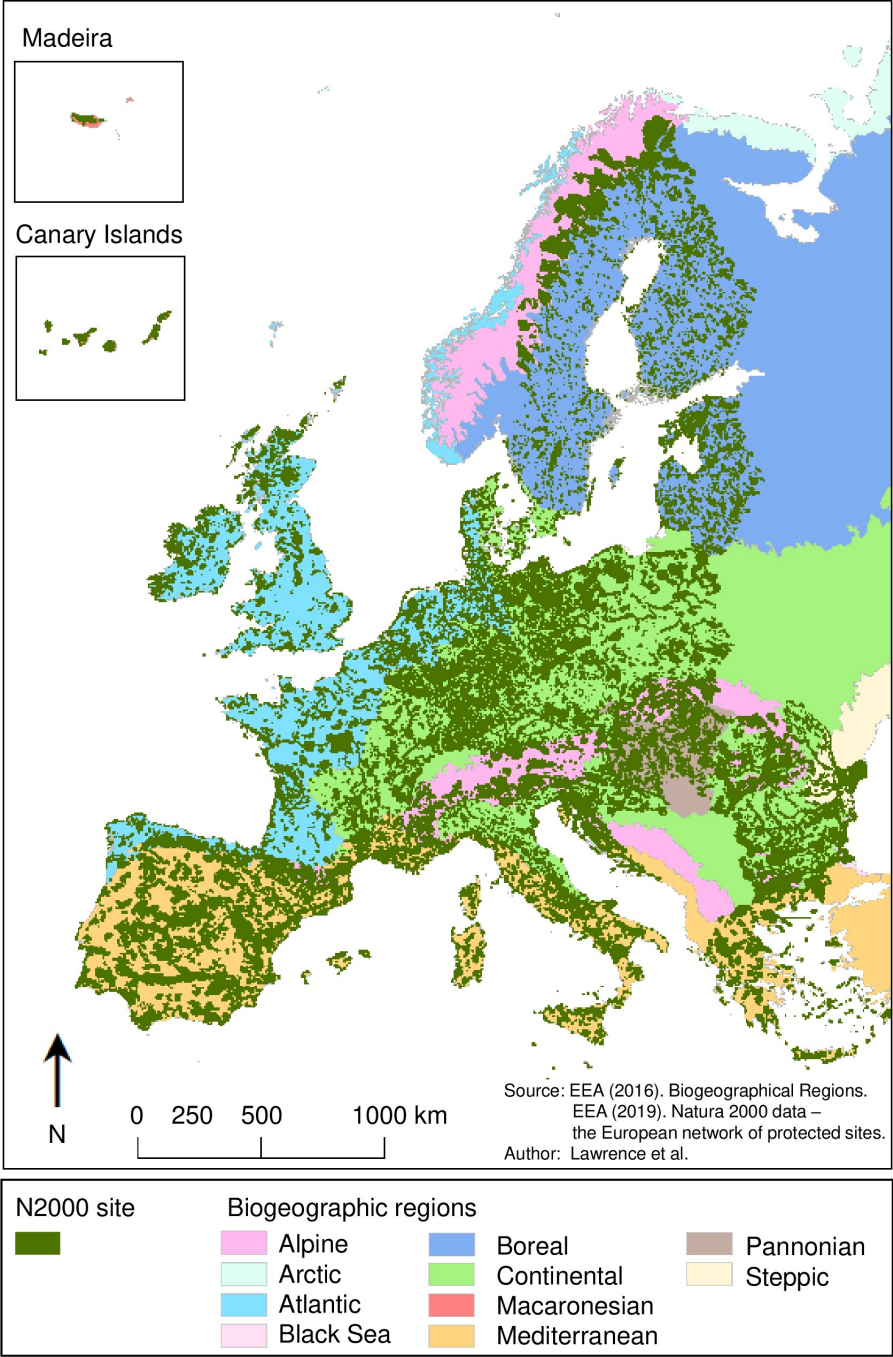
The European N2k network across nine biogeographical regions. Dark green polygons represent terrestrial N2k sites analysed in this study [47]. Map generated in ArcGIS 10.6.1 (http://www.esri.com/software/arcgis/arcgis-for-desktop).

This study quantifies landscape fragmentation within and around the European N2k network, the world’s largest coordinated network of PAs [15]. The N2k network spans 27 countries and nine different biogeographical regions (Fig 1).

Our analysis covers a total of 15390 terrestrial PAs that range in size from 1 km² to 5556 km² (Table 1) and range between 2 and 37 years in age. The N2k network is a heterogenous network of PAs that shows considerable differences in the distribution of PA numbers, sizes, ages, and relative area-coverage among the biogeographical regions (Table 1).

**Table 1:** The N2k network across biogeographical regions of the EU.

Biogeo-graphical region	Number of N2k PAs	Mean size of N2k PAs (km²)	Min-max. size of N2k PAs (km²)	Mean age of N2k PAs (years)	Relative number of N2k PAs	Relative area of N2k PAs
Alpine	1243	144	1–5556	19	8.1%	22.5%
Atlantic	2350	36	1–3465	22	15.3%	10.8%
Black Sea	48	209	1–4344	14	0.3%	1.2%
Boreal	2777	20	1–3095	19	18.0%	7.1%
Continental	5665	38	1–2915	18	36.8%	26.9%
Macaronesian	130	29	1–404	25	0.8%	0.5%
Mediterranean	2539	87	1–2186	22	16.5%	27.6%
Pannonian	568	41	1–1102	15	3.7%	2.9%
Steppic	69	66	1–577	12	0.4%	0.6%
Total	15390	52	1–5556	20	100%	100%

The distribution of N2k PAs in terms of number, size, age, and relative coverage by number and area varies considerably among biogeographical regions. The Alpine and the Black Sea regions host the largest N2k PAs by area, while the Continental region hosts the highest number of N2k PAs. “Age of N2k PAs”refers to the time since PAs first gained protected status. “Relative number of N2k PAs”refers to the number of N2k PAs within a biogeographical region relative to the total number of N2k PAs. “Relative area of N2k PAs”refers to the total area covered by N2k PAs within a biogeographical region relative to the total area covered by all N2k PAs.

### Effective mesh size and effective mesh density

In this study, we measured fragmentation by calculating effective mesh density, a landscape-scale metric developed by Jaeger [48]. Effective mesh density represents the degree of fragmentation in a landscape [48, 49]. Jaeger et al. [50] defined a series of ’fragmentation geometries’ (FGs) which include different types of barriers. This study focuses on major and medium anthropogenic constructions–such as roads, railways, and buildings. This coincides with fragmentation geometry A2 (FG-A2) described in more detail by Jaeger et al. [51].

To quantify fragmentation within a landscape, Jaeger [48] developed the landscape metric effective mesh size (*meff*), which is based on the probability that two points chosen randomly within a defined area will be connected (i.e., located in the same patch). This can be interpreted as the probability that two animals can find each other inside the defined area without crossing a barrier. Multiplying this probability by the total area of the area under study, it is converted into the size of an area: the effective mesh size. Hence, *meff* can be interpreted as the expected size of the area that is accessible for an individual animal from a randomly chosen point inside the defined area without encountering a barrier [48, 52].

As the number of fragmentation barriers increases, the mesh size diminishes, and therefore *meff* decreases in its value. If anthropogenic barriers cover a landscape entirely, *meff* has a value of 0 km². Originally, *meff* was calculated using the cutting-out (CUT) procedure. However, this method is affected by the boundary problem because the boundaries of the reporting units, e.g., the borders of raster cells, are considered additional barriers. To overcome this limitation, a new method called the "cross-boundary connections’’ (CBC) procedure attributes the connections between two points located in different reporting units to both reporting units. The CBC procedure is independent of the size and administrative boundaries of reporting units [52]. In this study, we used the CBC procedure.

Within a defined landscape–e.g., 1 km² grid cell–it is possible to calculate effective mesh density (*seff*), by taking the inverse of effective mesh size (*meff*) (Eq 1) [51].

$$seff=\frac{1}{meff}$$ (1)

The value of *meff* informs about the size of uninterrupted spaces and *seff* about the density of these uninterrupted spaces. Translated into an ecological context, the phenomena measured by effective mesh size and density impact the mobility of animals within a given range. Thus, this concept directly addresses landscape fragmentation and makes it possible to quantify the reduction in landscape connectivity [51].

### Data collection

In this study, we used a raster dataset showing the state of fragmentation in 2012/2014 for the European Union (Table 2). This dataset is based on 2012 Corine Land Cover (CLC) data and 2014 Teleatlas data and available on demand from the EEA [53]. Each raster cell has a resolution of 1 km^2^ and contains a value representing effective mesh size (*meff*) calculated via CBC procedure [52]. The administrative units refer to the 2016 Nomenclature of Territorial Units for Statistics (*Nomenclature des unités territoriales statistiques*, NUTS), which divides each EU Member State into three hierarchical regions. The data set comprises all countries at a 1:1 million scale. We used NUTS level 3 to investigate landscape fragmentation across the EU. Data on N2k sites encompass the Special Areas of Conservation (SAC) and the Special Protection Areas (SPAs) of the European Union, including the UK. In addition, each N2k site has information listed on its location within Europe’s biogeographical regions. To determine the age of N2k PAs, we used information provided by the World Database of Protected Areas (WDPA) (Table 2). From the WDPA database, only information from entries containing geographical data and reporting a PA’s protection status as "designated" was used.

**Table 2:** Processed data. In this study, we used open-access data to derive information on fragmentation within and around Natura 2000 (N2k) PAs, N2k PA locations within one of nine biogeographical regions, their locations within specific administrative units, and PA age.

Dataset	Information	Resolution	Date	Source	Open access
Fragmentation	Effective mesh size (*meff*) and density (*seff*)	1 km^2^	2012/2014	EEA [53]	Yes, on demand
Administrative units	NUT-3 regions	Vector data	2016	ESTAT [54]	Yes
Natura 2000	PA borders, PA size, biogeographical region	Vector data	2018	EEA [47]	Yes
WDPA	PA age	Vector data	2019	UNEP [55]	Yes

### Spatial data processing with GIS

All spatial data were processed using ESRI ArcGIS 10.6.1 and QGIS 2.18.25 in ETRS 89 Lambert Azimuthal Equal Area (LAEA) Projection. To calculate *meff* values for each N2k site, we first filtered the N2k site dataset to ensure each N2k covers at least one cell center of the *meff* raster dataset. To do so, we rasterized all N2k sites via cell center coverage using the 1-km^2^-resolution of the original *meff* dataset. This method entailed excluding N2k sites which did not cover at least one raster cell center. These were particularly small and elongated N2k sites. We also reduced our analysis to terrestrial PAs by excluding marine N2k sites. These exclusions resulted in a dataset comprised of 15390 N2k sites of the original 27845 N2k sites (55.3%).

We created a buffer zone of 5 km around each of these N2k sites. We further applied the same rasterization process to these buffer zones as to the N2k sites using cell center coverage based on the 1-km^2^-resolution of the original *meff* dataset. This approach has the advantage that each cell of the *meff* dataset is assigned only once and never to both a N2k site and a buffer. For fine-scale analyses focusing on the effects of PA surroundings on specific species, buffer size is often determined by migration or dispersal distances or habitat size requirements of those species [56]. However, as Holland et al. [57] have shown, the most appropriate spatial scale to analyse species’ responses to environmental variables varies tremendously between species even within the same family. As this is a landscape-focused study, the chosen buffer size does not consider species-specific indicators and instead is homogenous for all N2k sites. Still, it is nearly impossible to determine a uniform buffer size best suited to analyse anthropogenic fragmentation around a PA relative to its interior. The most appropriate buffering distance varies according to location and the conservation focus of each PA. In this study, we consider a 5 km buffer around each N2k site following previous studies investigating landscape factors such as surrounding agriculture [58] and surrounding land cover changes [59]. As in those studies, our focus is on surrounding areas close to N2k sites, rather than comparing them against distant areas which presumably have a less immediate influence on ecosystems inside PAs.

To calculate effective mesh size (*meff*) and effective mesh density (*seff*) for each N2k site individually, we first summed up the *meff* values of all grid cells (1-*n*), the cell centers of which fell within the N2k site. Since *meff* is area-proportionately additive [52], we calculated an individual *meff* value for each N2k site by dividing the sum of *meff* values within the N2k site by *n*, the total number of cells within the N2k site (Eq 2).

$$meff=\frac{\sum_{i=1}^{n}meff_{i}}{n}$$ (2)

Taking the inverse of the new site-specific *meff* value resulted in one site specific *seff* value (Eq 1). *seff* values represent the number of meshes per km^2^. In order to follow EEA standards, we report final *seff* values in meshes per 1000 km^2^ [51]. We used the same steps as described above for determining *seff* values for the 5 km buffer zone around each N2k site.

We further calculated the median *seff* value of N2k sites and of their surroundings for each NUTS-3 region. Obtaining the median fragmentation value per NUTS-3 region allowed us to present the data on a broader spatial scale compared to presenting the data for each N2k PA individually (S1 Fig). This presentation also allowed for better visual comparison of regions within the EU relative to the presentation of raw data. The median instead of the mean was used to lessen the impact of outliers. It is important to consider that one large fragmented N2k site has the same influence on the median of the NUTS-3 region as a small N2k site. For more detailed information on individual N2k sites, we published our raw data (https://doi.org/10.6084/m9.figshare.13513902) and added S1 Fig to the supporting information. To minimize information loss, statistical analysis was performed exclusively on raw data instead of using medians.

Many of the N2k sites were already protected prior to the establishment of the N2k network in 1992. We used each site’s age since first designation as a PA. To determine the age of N2k sites, data on protected areas from the World Database of Protected Areas (WDPA) [55] were intersected with N2k data. N2k sites commonly overlap with other protected areas, such as national parks or biosphere reserves. In case a single N2k site overlapped with several protected areas, the earliest date of designated protection was used to calculate the age of the N2k site. Of our total 15390 N2k sites, we identified the age for 15335 N2k sites (99.6%).

### Statistical analysis

Statistical analysis was performed in R v. 3.6.2 [60]. We assigned each N2k site and each 5 km buffer zone to one of five fragmentation categories following previous EEA reports [46] (Table 3). This categorization was used primarily for the visualization of the data.

**Table 3:** Categories of effective mesh density (*seff*). In contrast to the EEA [46], the fragmentation categories "anthropogenic" and "very high" were combined into one category “very high” due to the relatively small number of N2k sites within the anthropogenic category.

Effective mesh density (number of meshes per 1000 km²)	Fragmentation Category
≤ 1.5	Very low
> 1.5–10	Low
> 10–50	Medium
> 50–250	High
> 250	Very high

We conducted linear regressions using the lm function to examine the relationship between fragmentation of N2k sites and their surroundings. The effective mesh density of the surroundings of N2k sites (*seff*_*surrounding*_) served as the predictor variable and the effective mesh density within the N2k sites (*seff*_*within*_) as the response variable. The data were log (x+1) transformed to meet normality requirements. We also created nine individual linear regression models relating *seff*_*surrounding*_ and *seff*_*within*_ to the nine biogeographical regions covered by N2k sites. To analyse the relationship between the area of N2k sites and fragmentation within N2k sites, we conducted linear regressions using N2k site area as the predictor and *seff*_*within*_ as the response variable.

To test for correlation between site age and the difference of fragmentation within and around N2k sites, we first calculated the difference between mesh density inside a N2k site (*seff*_*within*_) and outside a N2k site (*seff*_*surrounding*_) by subtracting *seff*_*within*_ from *seff*_*surrounding*_ for each N2k site separately (Eq 3).

$$seff_{diff}=seff_{surrounding}-seff_{within}$$ (3)

We then conducted linear regression using age as predictor and *seff*_*diff*_ as response variable. For all linear regressions, the assumptions of linear regressions were verified by using diagnostic plots (*plot(linear model*)) showing linearity, homoscedasticity, and no substantial influence of extreme values.

## Results

### Fragmentation within and around N2k sites

**Fig 2:**
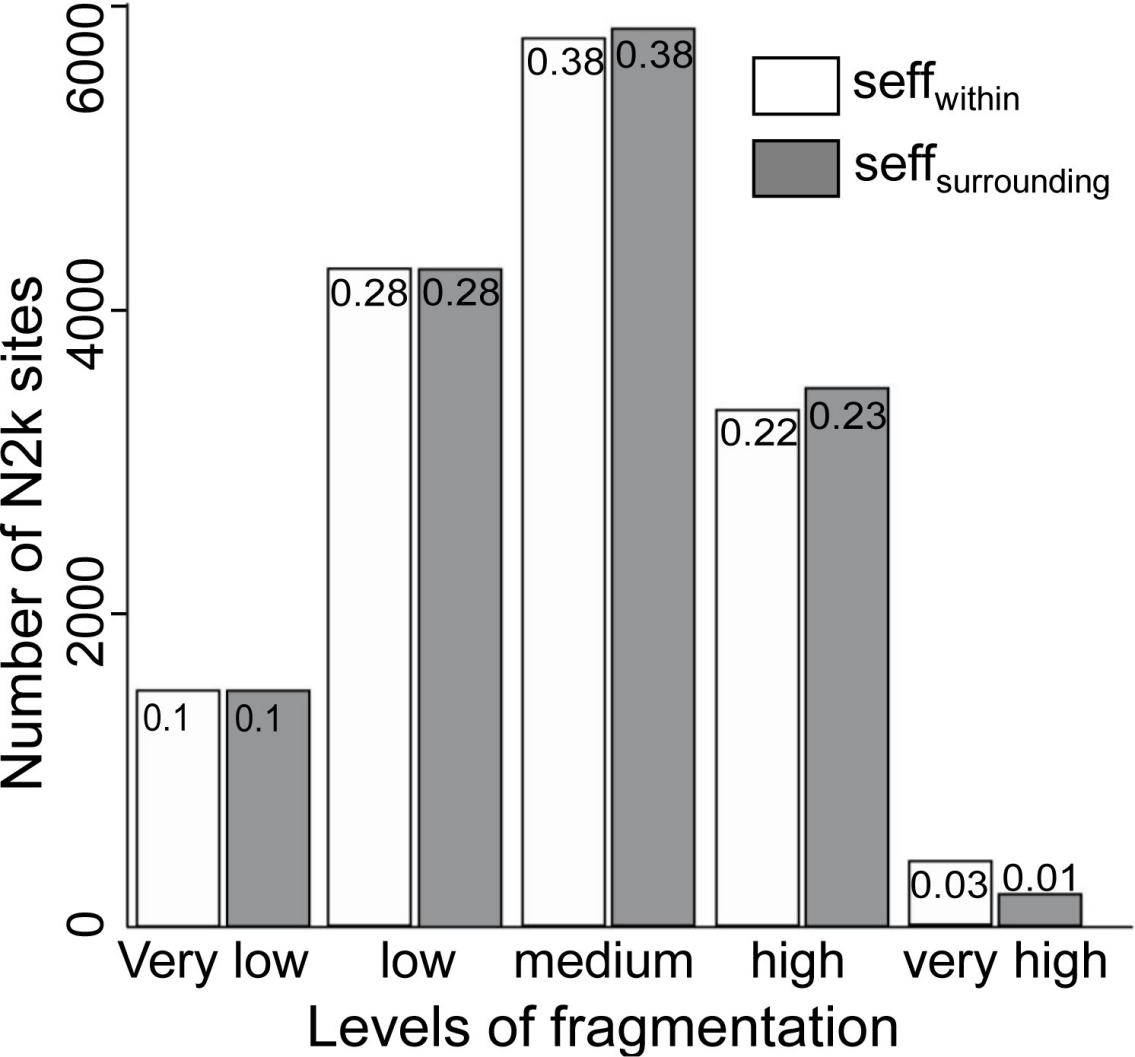
Number of N2k sites and their surroundings per fragmentation category. Fragmentation categories are based on EEA standards for effective mesh density (Table 3). Decimal numbers represent the relative amount of N2k sites which fall in one of the five fragmentation categories. The total number of N2k sites analyzed was 15390 (55.3% of N2k sites).

Based on absolute *seff* values, 58.5% of all N2k sites are less fragmented than their surroundings, 0.9% are equally fragmented, and 40.6% are more fragmented than their surroundings. When categorized according to EEA standards (Table 3), N2k sites exhibit all levels of fragmentation from very low to very high (S1 Fig). Most N2k sites and their surroundings show a medium level of fragmentation, and only a few N2k sites and their surroundings show very low or very high levels of fragmentation (Fig 2). Surroundings of N2k sites are mostly within the same fragmentation category as N2k sites themselves (Fig 2).

### Fragmentation patterns of N2k sites across the EU

**Fig 3:**
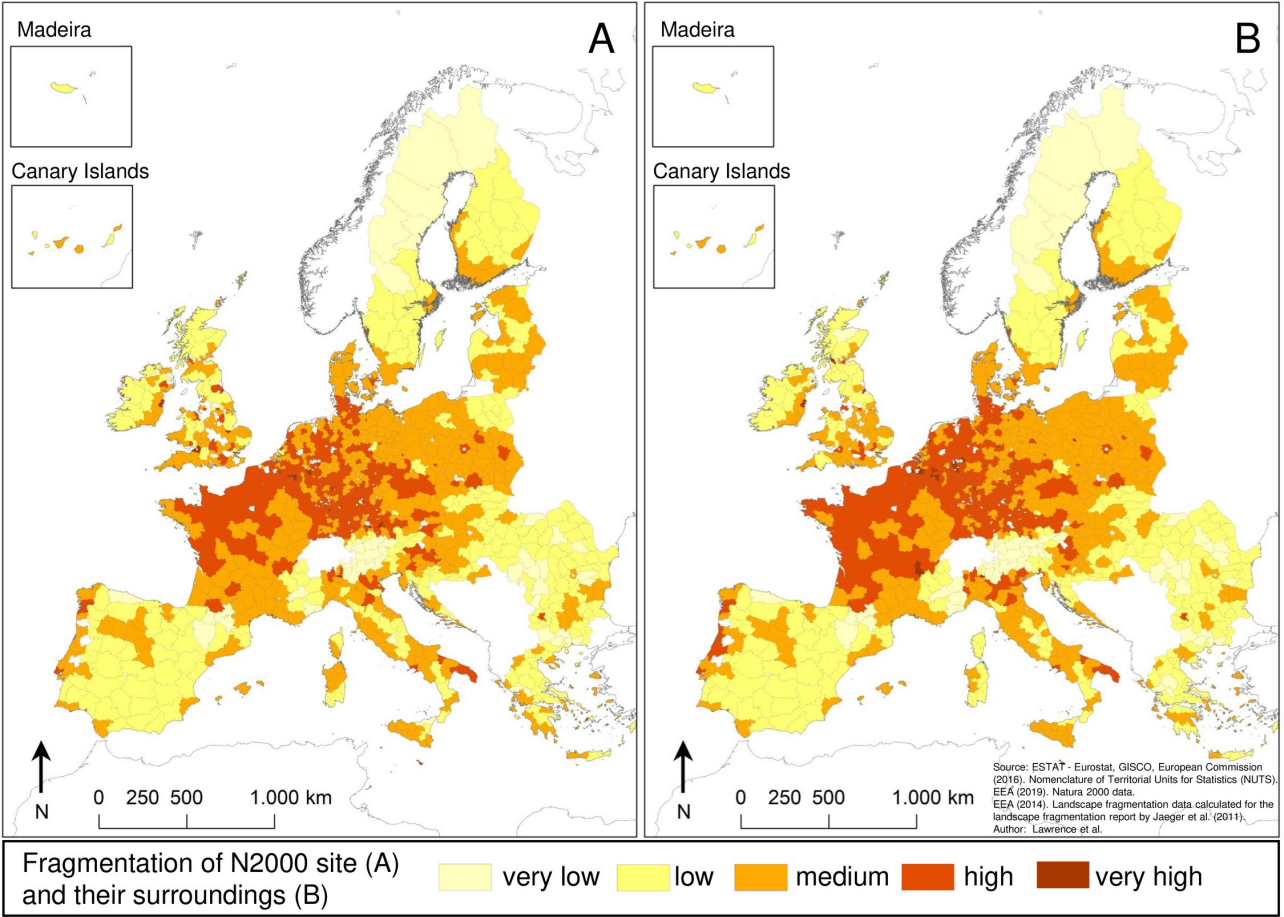
Fragmentation within N2k sites (*seff*_*within*_) (A) and in their surroundings (*seff*_*surrounding*_) (B) for NUTS-3 regions. The value for each NUTS-3 region was obtained by calculating the median of *seff*_*within*_ (A) and the median of *seff*_*surrounding*_ (B) of N2k sites for each NUTS-3 region. The area-wide coloration on the map reflects only the degree of fragmentation within (A) or in the surroundings of (B) N2k sites rather than the area-wide fragmentation throughout the NUTS-3 regions. Map generated in ArcGIS 10.6.1 (http://www.esri.com/software/arcgis/arcgis-for-desktop).

All five fragmentation categories are represented when taking the median for *seff*_*within*_ (Fig 3A) and *seff*_*surrounding*_ (Fig 3B) for each NUTS-3 region. Fragmentation inside and outside N2k sites is highest in central Europe, especially in France, Belgium, the Netherlands, Luxembourg, Germany, and the Czech Republic (Fig 3A and 3B). N2k sites and their surroundings showing very low levels of fragmentation are predominantly located in remote and/or mountainous regions of the EU, such as large parts of Sweden, Finland, Romania, the border between Bulgaria and Greece, the French, Italian and Austrian Alps, and the French and Spanish Pyrenees (Fig 3A and 3B). For most NUTS-3 regions, the category of interior fragmentation does not differ from the category of fragmentation for N2k sites’ surroundings. Some NUTS-3 regions in France, Italy, and along the coast in Portugal show lower fragmentation categories inside N2k sites (Fig 3A) compared to N2k surroundings (Fig 3B).

**Fig 4:**
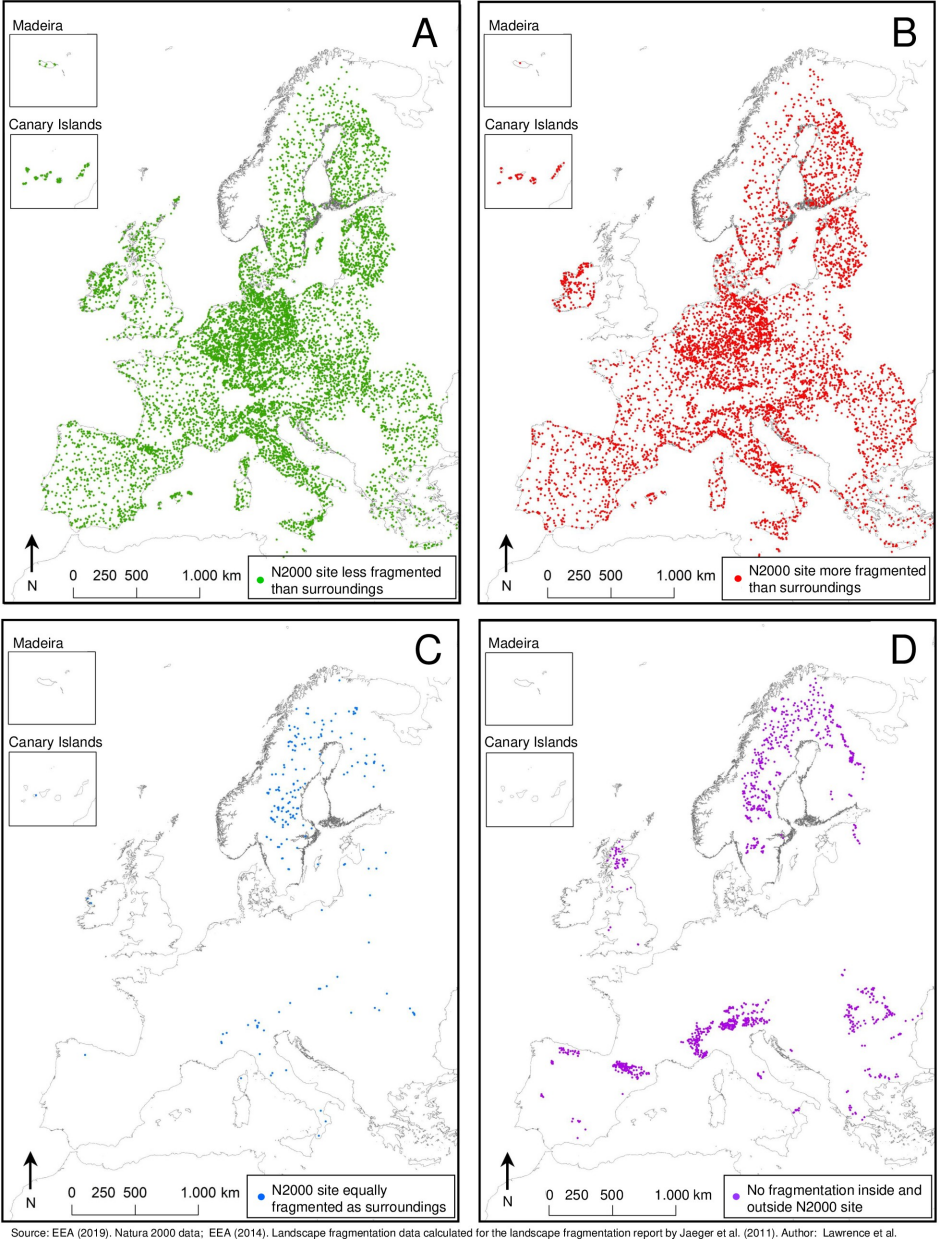
Fragmentation difference between N2k sites and their surroundings. Each point represents the centroid of a N2k site: When calculating *seff*_*diff*_ = *seff*_*surrounding*_—*seff*_*within*_, 58.5% of N2k sites are less fragmented than their surroundings (*seff*_*diff*_ > 0 meshes per 1000 km^2^) (A); 40.6% of N2k sites are more fragmented than their surroundings (*seff*_*diff*_ < 0 meshes per 1000 km^2^) (B); 0.9% of N2k sites are equally fragmented as their surroundings (*seff*_*diff*_ = 0 meshes per 1000 km^2^) (C); and independent from *seff*_*diff*_, no or only marginal fragmentation within and in the surrounding of N2k sites (*seff*_*within*_ and *seff*_*surrounding*_ ≤ 0.9 meshes per 1000 km^2^) exists for 6.9% of N2k sites (D). Map generated in ArcGIS 10.6.1 (http://www.esri.com/software/arcgis/arcgis-for-desktop).

According to the absolute difference between *seff*_*surrounding*_ and *seff*_*within*_ (Eq 3), 58.5% of N2k sites are less fragmented than their surroundings and those sites are distributed throughout the EU (Fig 4A). Similarly, N2k sites which are more fragmented than their surroundings (40.6%) are also represented throughout the EU (Fig 4B). N2k sites for which *seff*_*within*_ and *seff*_*surrounding*_ are exactly equal are rare (0.9%) and mainly located in northern Europe, the Alps, and parts of Romania (Fig 4C). N2k sites that exhibit no or only marginal fragmentation inside their boundaries as well as in their immediate surroundings (*seff*_*within*_ and *seff*_*surrounding*_ ≤ 0.9 meshes per 1000 km^2^) (6.9%) are almost exclusively found in remote and mountainous regions of the EU (Fig 4D). This includes large parts of Sweden, Finland, parts of Romania, the French, Italian and Austrian Alps, the French and Spanish Pyrenees, and parts of the Scottish Highlands.

### Size and area coverage of fragmented N2k sites

**Fig 5:**
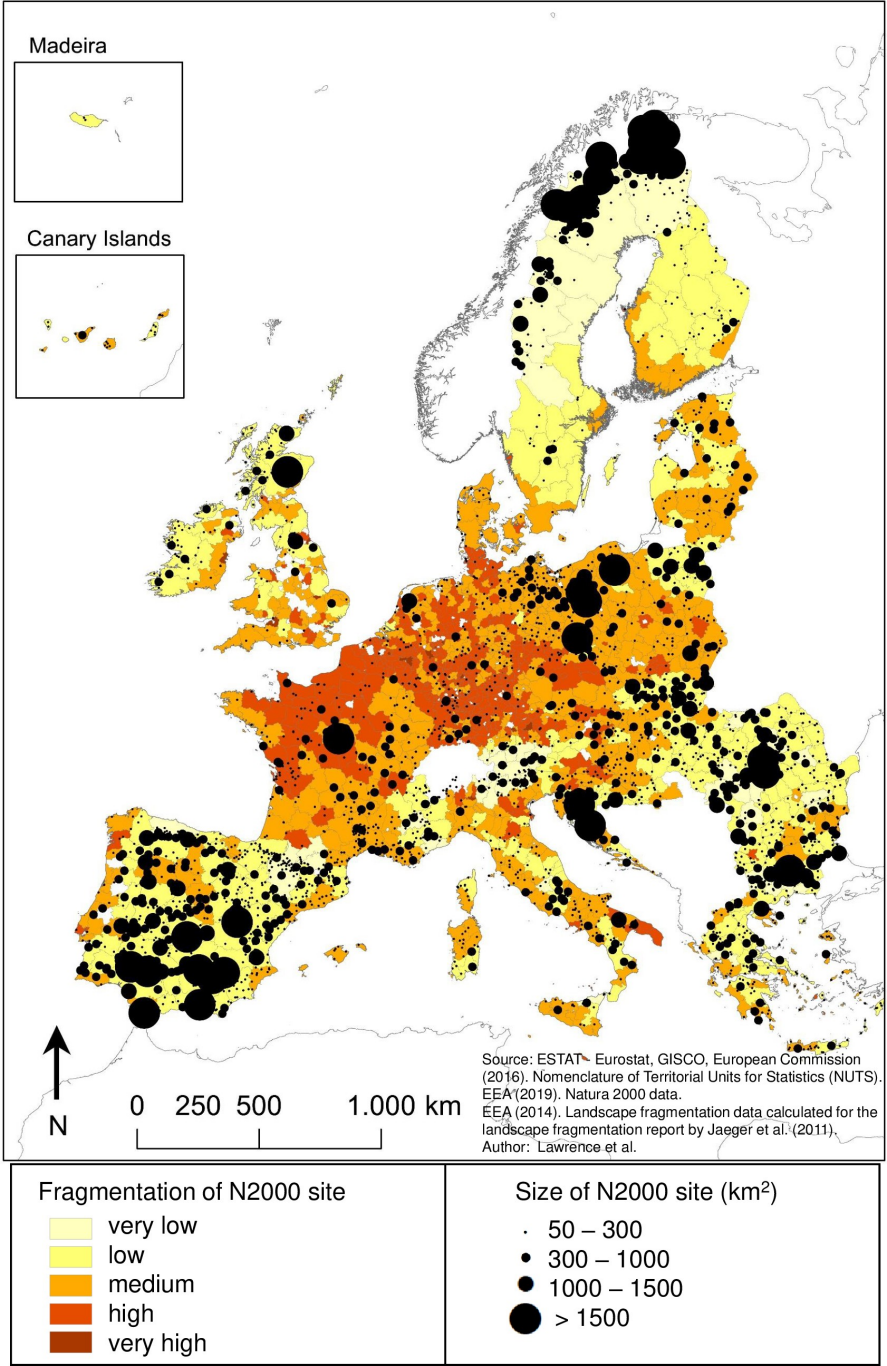
Size of N2k sites and fragmentation within N2k sites (*seff*_*within*_). The value for each NUTS-3 region was obtained by calculating the median of *seff*_*within*_ of N2k sites for each NUTS-3 region. The area-wide coloration on the map reflects only the degree of fragmentation within N2k sites rather than the area-wide fragmentation throughout the NUTS-3 regions. Large N2k sites are predominantly located in remote and mountainous regions, which tend to exhibit low fragmentation. Map generated in ArcGIS 10.6.1 (http://www.esri.com/software/arcgis/arcgis-for-desktop).

The sizes of the N2k sites analysed in this study vary greatly between the smallest (1 km²) and largest site (5556 km²). The majority (90.1%) of all N2k sites cover an area between 1 km² (5% quantile) and 243 km² (95% quantile). Large N2k sites are mainly located in remote and mountainous regions of the EU (Fig 5). N2k sites larger than 1500 km^2^ are found predominately north of the polar circle in Sweden and Finland, in eastern Europe in parts of Poland, Romania and the Balkans, as well as in southern Spain (Fig 5). N2k area and fragmentation within N2k sites (*seff*_*within*_) are negatively correlated (R^2^ = 0.09, p < 0.001).

**Fig 6:**
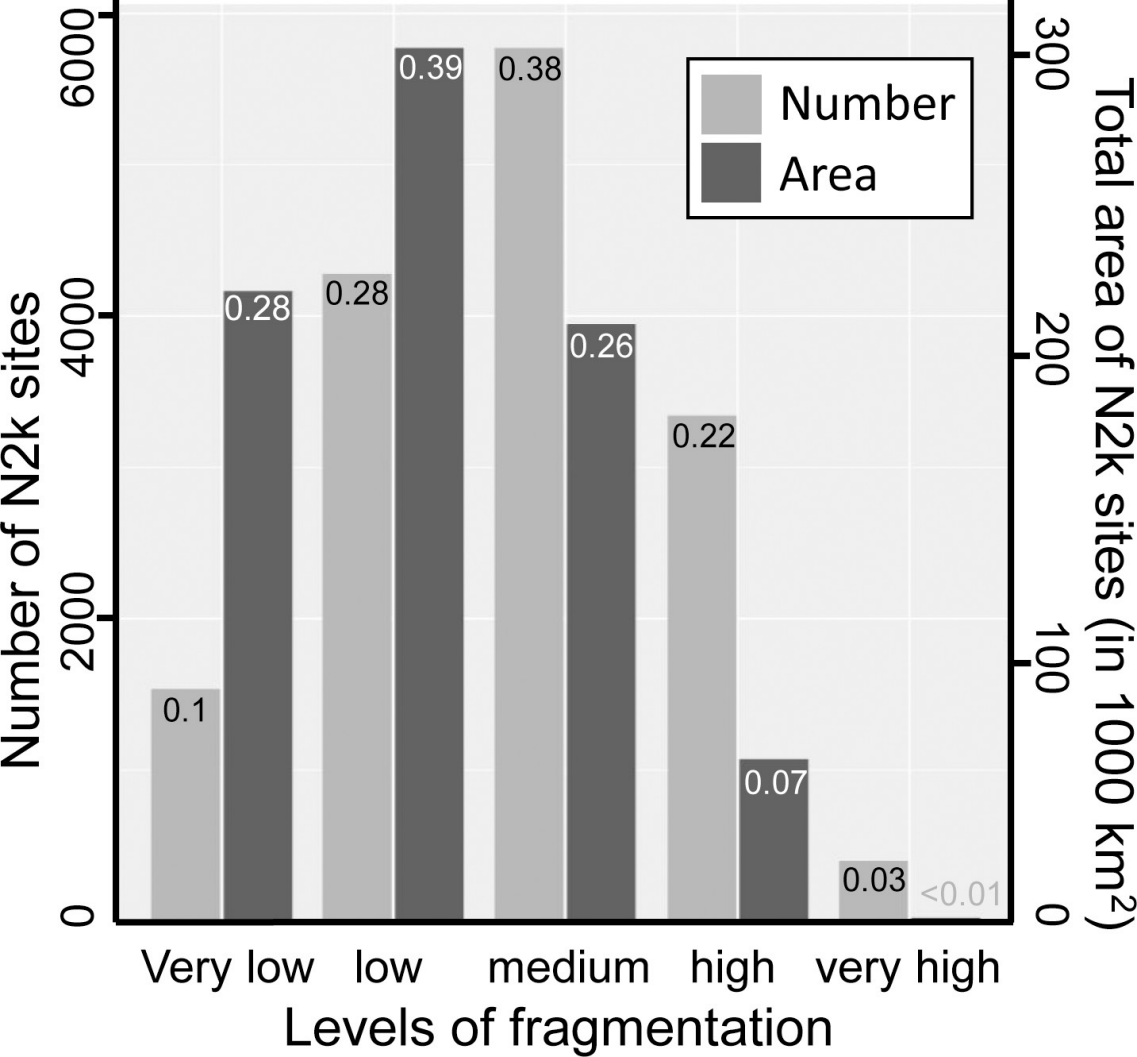
Number and area coverage of N2k sites per fragmentation category. Bars in light grey represent absolute numbers of N2k sites analyzed in this study. Bars in dark grey represent absolute area coverage in 1000 km^2^ of N2k sites analyzed in this study. Black decimal numbers represent the relative amount of sites for each of the five fragmentation categories. White decimal numbers represent the relative amount of area covered by N2k sites for each of the five fragmentation categories. The total number of N2k sites analyzed was 15390 (55.3% of N2k sites). The total area covered by N2k sites analyzed was 796637 km^2^ (79.5% of the total area covered by N2k sites). Fragmentation categories are based on EEA standards for effective mesh density (Table 3).

We also compared the number of N2k sites to the cumulative area covered by N2k sites for the different fragmentation categories (Fig 6). N2k sites of low and very low fragmentation are few in number (37.9%) but they cover over 66.3% of the total area within the N2k network analyzed in this study (Fig 6). In contrast, N2k sites that show fragmentation levels in the categories medium, high, and very high, amount to 62.1% of the total number of N2k sites analyzed but cover only 33.7% of the cumulative area of N2k sites analyzed in this study.

### Interior and exterior fragmentation of N2k sites for the biogeographical regions of the EU

**Fig 7:**
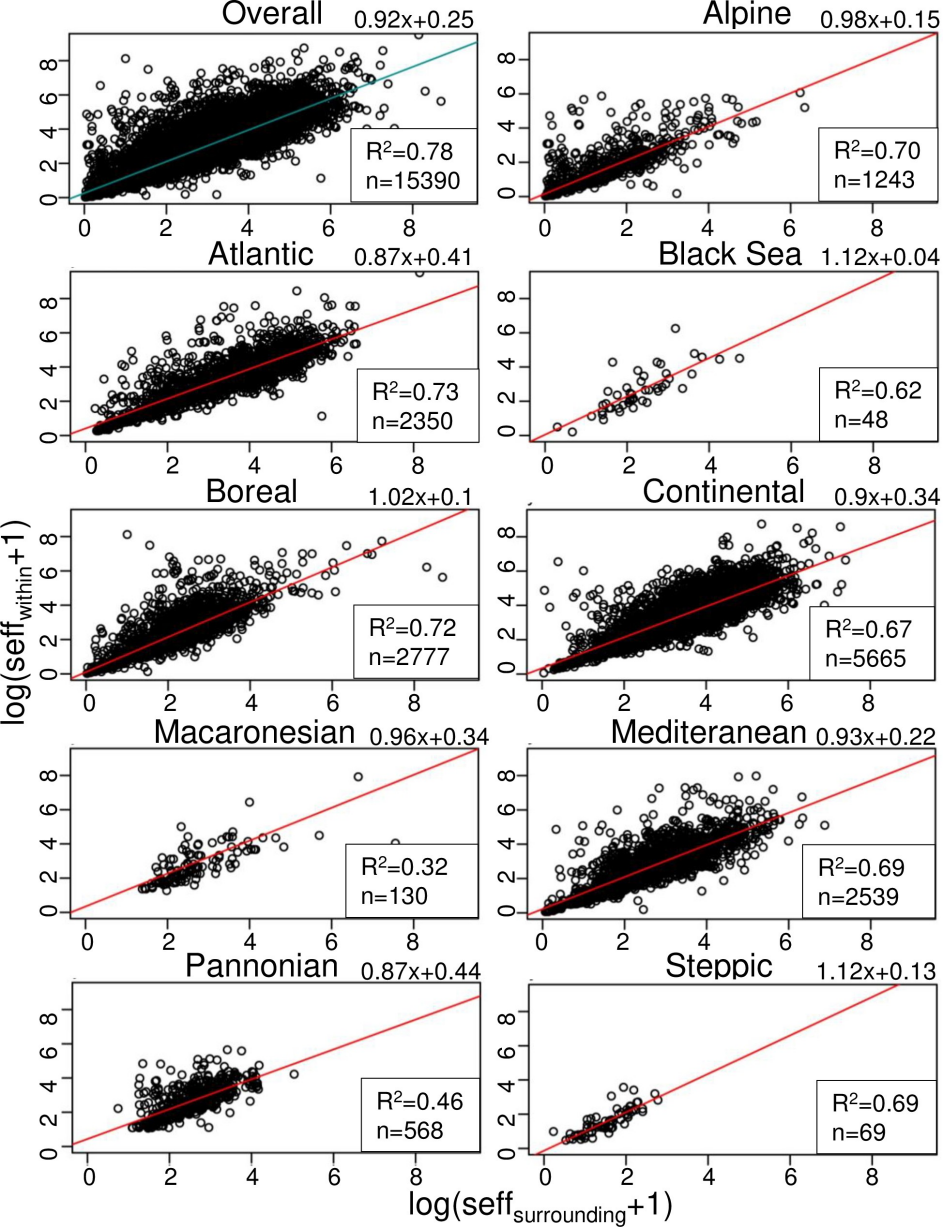
Correlation between effective mesh density (*seff*) within and around N2k sites for the nine biogeographical regions of the EU. *seff*_*within*_ significantly correlates with *seff*_*surrounding*_ for all nine biogeographical regions. For each biogeographical region, the *R*^*2*^ value, the number of sites (*n*), and the linear regression formula with *x = seff*_*surrounding*_ are provided.

Linear regression was used to predict interior fragmentation of N2k sites (*seff*_*within*_) based on the fragmentation of their surroundings (*seff*_*surrounding*_). The results show a strongly significant relationship between *seff*_*surrounding*_ and *seff*_*within*_ (*seff*_*within*_ = 0.9 *seff*_*surrounding*_ + 0.3, p < 0.001, R² = 0.78) (Fig 7). We also analysed the relationship between *seff*_*within*_ and *seff*_*surrounding*_ for each biogeographical region separately using linear regressions. Our results demonstrate that *seff*_*within*_ significantly increases with increasing *seff*_*surrounding*_ for all nine biogeographical regions (Fig 7). *seff*_*surrounding*_ explains 67% or more of the variance observed in *seff*_*within*_ for all biogeographical regions, except for the Black Sea (R^2^ = 0.62), Macaronesian (R^2^ = 0.32) and Pannonian (R^2^ = 0.46) regions, which are also characterized by relatively small sample sizes (Fig 7). For all nine biogeographical regions, except for the Black Sea, Boreal, and Steppic region, the regression slope is slightly below 1, i.e., an increase by 1 mesh per 1000 km^2^ in *seff*_*surrounding*_ results in an increase of slightly less than 1 mesh per 1000 km^2^ in *seff*_*within*_. For the Black Sea, Boreal, and Steppic regions, the regression slope is slightly above 1. This means that an increase in *seff*_*surrounding*_ by 1 mesh per 1000 km^2^ results in an increase of slightly more than 1 mesh per 1000 km^2^ in *seff*_*within*_ (Fig 7).

### Difference in interior and exterior fragmentation of N2k sites in relation to site age

**Fig 8:**
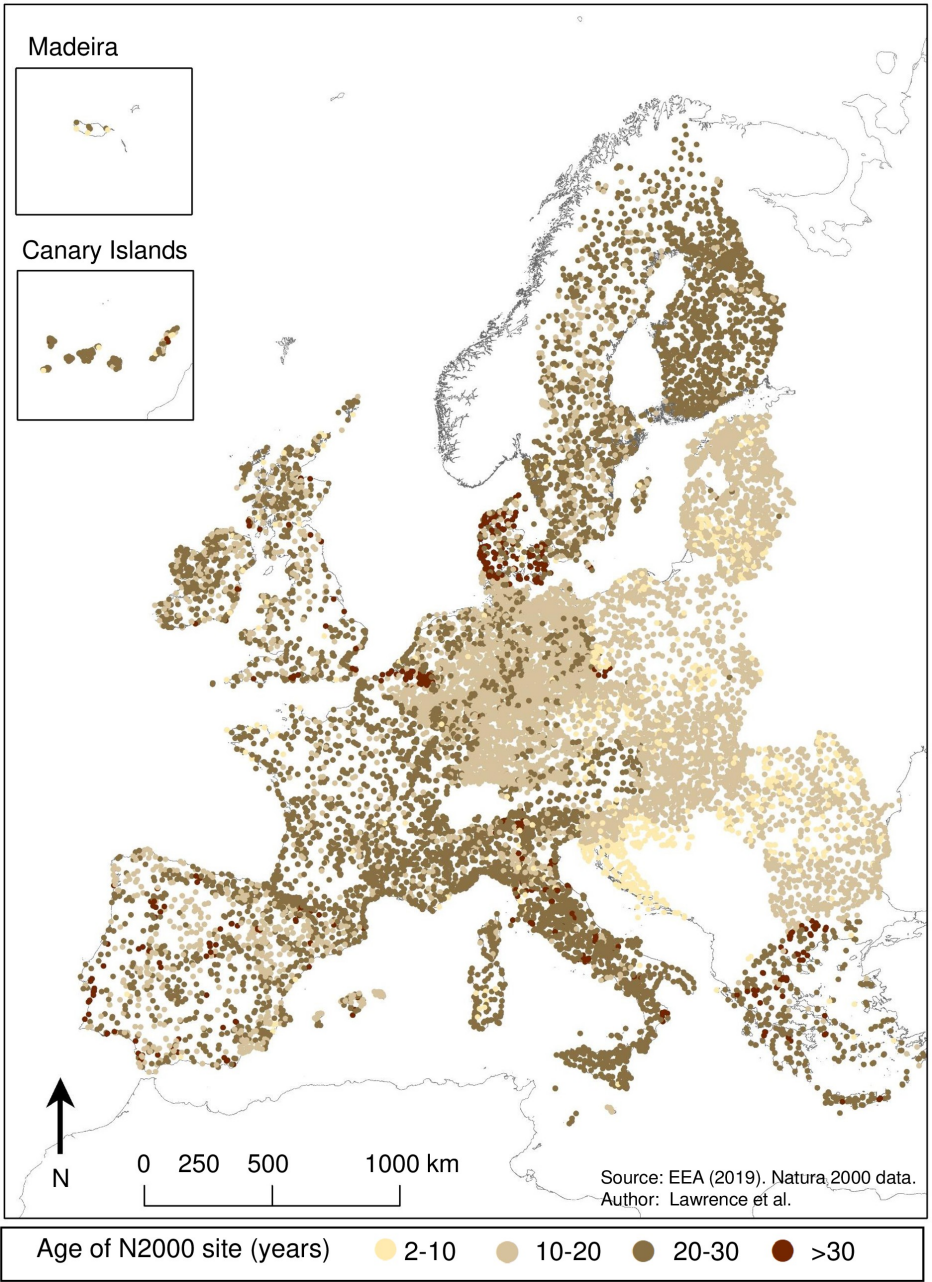
Age of N2k sites. Recently protected PAs within the N2k network are predominantly located in eastern European member states which joined the EU since 2004 (Status 2017) [55]. Map generated in ArcGIS 10.6.1 (http://www.esri.com/software/arcgis/arcgis-for-desktop).

N2k sites analysed in this study differed in age between 2 and 37 years. Young N2k sites are predominantly located in eastern European member states, especially Croatia, which joined the EU in recent years. In contrast, the oldest N2k sites are predominantly located in Spain, France, Italy, Greece, Denmark, and the Netherlands (Fig 8).

There is no significant correlation between N2k age and the absolute difference in effective mesh density (*seff*_*diff*_) within and around N2k sites (R2 < 0.001, p = 0.58). In other words, N2k sites are not significantly less fragmented compared to their surroundings the longer their protected status has been in place. This result contradicts our original hypothesis.

## Discussion

Despite their protected status, our results show that N2k sites are very fragmented. Moreover, fragmentation within N2k sites strongly correlates with the fragmentation of their surroundings. This correlation applies to all nine biogeographical regions in the EU. Remote and mountainous regions show the lowest levels of fragmentation within and around N2k sites. These remote and mountainous regions also tend to hold the largest N2k sites by area. Further, N2k PA age does not correlate with the difference in exterior and interior fragmentation of N2k PAs. Our results suggest that there is high potential for improving PA efficacy by taking pre-emptive action against encroaching anthropogenic fragmentation and by targeting scarce financial resources in nature conservation where fragmentation pressures can be mitigated feasibly through enforced construction bans inside PAs.

### Limitations to analysing fragmentation of N2k sites

While N2k sites form a network of PAs designated for nature conservation and biodiversity preservation, each site’s conservation value is largely based on land use history. These local histories can include long periods of anthropogenic changes as in areas formerly exploited for agricultural production or resource extraction. The biota that have established and evolved within these cultural landscapes are no less crucial for biodiversity compared to those that have evolved within landscapes which have experienced little or no anthropogenic change [16, 59]. Linear structures such as historic roads or canals are intrinsic features of many N2k sites that host high numbers of species adapted to these unique habitats [61]. Therefore, it is to be expected that some degree of fragmentation exists within much of the N2k network. However, these land use histories do not account for the high degree of fragmentation we found within N2k sites. Economic development is the main driver of European landscape fragmentation [51], and our results suggest that N2k sites are not sufficiently sheltered from contemporary fragmentation pressures.

While our data show that the vast majority of N2k sites (93.1%) are fragmented to some degree, limitations in our study design led to the exclusion of a large number of sites from our analysis. We used pre-processed data on the effective mesh size (*meff*) and effective mesh density (*seff*), with a resolution of 1 km². However, it was necessary to apply a filter to ensure that each N2k site analysed covers at least one 1 km^2^ raster cell-center containing information on *meff* and *seff*. This led to the exclusion of several small or elongated N2k sites. Despite their requisite exclusion, these small N2k sites are an important component of the N2k network. They can be vital habitats for confined or for small-range species, or they may contribute to landscape complementation and overall habitat diversity [9, 45]. Further, small N2k sites are unevenly distributed across Europe [62] and might therefore play an outsized role in nature conservation for some countries relative to others. The range and distribution of differently-sized PAs within the N2k network is a result of political considerations, societal criteria, and the regional patterns of high conservation value habitats [63–65]. There is no single standard criterion that was applied in the designation of N2k sites across Europe [65]. As a consequence, the regional and biogeographical specifics in PA size, naturalness, as well as in fragmentation, are simultaneously constraints and inherent qualities of the N2k network. With additional research quantifying the degree of fragmentation for small N2k sites—by, for example, calculating *meff* and *seff* directly using OpenStreetMap (OSM) data [66],—we could gain a comprehensive understanding of fragmentation pressures posed to the N2k network as a whole and better design anti-fragmentation management plans at the national level.

### Anthropogenic fragmentation inside N2k sites

Previous studies have demonstrated the N2k network’s low effectiveness in protecting certain target species [67–69]. Our study is the first to quantify fragmentation differences between the interior of N2k sites and their surroundings, and our results suggest that failure to account for fragmentation in and around N2k sites may contribute to these sites’ subpar effectiveness. Fragmentation within N2k sites strongly correlates with the fragmentation of their surroundings (R² = 0.78). Indeed, only a narrow majority of N2k sites are less fragmented than their surroundings.

We hypothesized the difference between exterior and interior fragmentation of N2k PAs to be bigger the longer protected status has been in place, reflecting effective PA management even if anthropogenic fragmentation in PA surroundings continues unabated. However, contrary to our original hypothesis, no correlation between PA age and the difference of fragmentation outside and inside the PA (*seff*_*diff*_) was found. It is possible that too little time has passed since PAs within the N2k network gained PA status for protection to have had a measurable effect in mitigating fragmentation pressures. The oldest PAs tested are no more than 37 years old and many N2k sites have only recently acquired protected status. This is especially true for N2k sites located in eastern European member states such as Bulgaria, Czech Republic, Estonia, Hungary, Latvia, Lithuania, Poland, Romania, Slovakia and Slovenia, which have been members of the EU only since 2004 [70]. It will be interesting to see if and how differences between fragmentation of PAs and their matrix do or do not change over time. In general, eastern European countries have lower population densities, have experienced delayed economic development, and have sparser road networks compared to central or western Europe. However, transportation infrastructure and urbanization have developed quickly in these countries since their accession to the EU [35]. This rapid development is likely to result in conflicts with conservation objectives in the future [16]. Therefore, we recommend a continuous monitoring of fragmentation within and around N2k PAs.

With a well-enforced nature conservation strategy, it should be possible to shelter existing N2k sites from encroaching anthropogenic fragmentation. Spatial concepts and priorities need to be developed in due course. This does apply not only to eastern Europe but to the entire EU. Our study provides information on the location of N2k sites most threatened from fragmentation. These results can be combined with projections of future economic development and projections of PA climate sensitivity to develop criteria for priority areas where conservation resources can be most efficaciously applied. This can provide a roadmap for N2k planners to ensure the continued viability of the crucial ecosystems located within the current N2k network. To shelter N2k sites from the deleterious effects of habitat loss and fragmentation, we suggest, based on our results: a) inscribing a ban of additional development inside N2k sites into law; b) putting additional resources into enforcing bans on constructions inside N2k sites; and c) where possible, removing extant fragmenting infrastructure.

### Distribution of low-fragmented N2k sites and implications for future conservation strategies

Our data show that large N2k sites of low fragmentation tend to be located where topography limits human infrastructure development. One reason for this phenomenon might be that establishing PAs in sparsely populated, mountainous, and far-northern landscapes is often easier and cheaper than in alternative locations. There is typically less pressure to use this land for agriculture or urban expansion. Low fragmentation is mainly found in remote regions, such as the Black Sea, Steppic, Macaronesian, Pannonian, or Boreal regions, as well as in mountainous regions such as the Alpine region. In fact, many N2k sites in mountainous regions have escaped anthropogenic fragmentation altogether, within and around N2k sites, highlighting the effect of topographical constraints.

At the country level, Sweden, Finland, and Romania manifest low or very low levels of fragmentation. However, in contrast to Sweden and Finland, Romania is in the process of expanding its road and rail infrastructure which will increase fragmentation in the future [51]. Some of this infrastructure may cut through existing PAs and will likely increase habitat fragmentation and species population decline, threatening–among others—the survival of several large mammal populations protected under the Habitats Directive such as bears, wolves, and lynx [51, 71]. Given the importance of Romania’s ecosystems for European biodiversity, Romania’s relatively untouched N2k sites need careful monitoring and active management to protect them from future fragmentation. One existing legal framework for this effort is the Carpathian Convention (2003), which explicitly addresses regulations on traffic and development [35]. We argue that similar legal concepts addressing anthropogenic fragmentation should be incorporated into N2k management in order to strengthen its long-term viability for continent-wide biodiversity conservation.

Target 11 of Aichi Biodiversity Targets, which aims to protect 17% of terrestrial area in each signatory country, results in a common and politically expedient conservation strategy of protecting those areas in which political and economic development pressures are weakest [21]. Protecting areas in marginal lands (also referred to as "rock and ice" [21]) to avoid competition with other economic and societal interests is a popular and politically viable way for countries to achieve their 17% target. This strategy is based on political expediency rather than science-backed conservation goals such as minimizing biodiversity loss. Given the essential contribution of smaller and often more fragmented PAs to biodiversity [9], this "rock and ice" strategy is dangerously inadequate as a conservation policy without including scientific findings on effective biodiversity preservation. However, our research suggests that strict enforcement of anti-fragmentation policies, such as those outlined above, can have immense benefits in those large, remote, and mountainous PAs that are already protected. These benefits derive from the fact that non-arable and sparsely populated areas, such as far northern or mountainous landscapes, are not well-suited to anthropogenic land use today, but economic and development interests of these areas may expand as a result of climate change. Therefore, today’s politically expedient solution of conserving large sections of remote, low-fragmented areas can, if strictly maintained and enforced, help prevent fragmentation pressures from undercutting conservation efforts in the future. By focusing on enforcement of construction bans inside PAs as an inexpensive and comparatively uncontroversial strategy, conservationists can take this opportunity to guard PAs against future anthropogenic pressure.

## Supporting information

S1 Fig Fragmentation within N2k sites (A) and in their surroundings (B). We calculated *seff*_*within*_ (A) and *seff*_*surrounding*_ (B) for each N2k site. The coloration of N2k sites represents one of five fragmentation categories (Table 3). Map generated in ArcGIS 10.6.1 (http://www.esri.com/software/arcgis/arcgis-for-desktop).
(TIF)

## References

[1] Millenium Ecosystem Assessment. 2005. Ecosystems and human well-being: Biodiversity synthesis. World resources institute, Washington, DC, USA.

[2] IUCN. IUCN Red List of Threatened Species. IUCN, Gland, Switzerland. 2010.

[3] Cardinale BJ, Duffy JE, Gonzalez A, Hooper DU, Perrings C, Venail P, et al. Biodiversity loss and its impact on humanity. Nature. 2012;486(7401): 59–67. doi: 10.1038/nature11148 22678280

[4] Crooks KR, Burdett CL, Theobald DM, King SRB, Di Marco M, Rondinini C, et al. Habitat fragmentation and extinction risk. Proceedings of the National Academy of Sciences. 2017;114(29): 7635–7640. doi: 10.1073/pnas.1705769114 28673992 PMC5530695

[5] Barnosky AD, Hadly EA, Bascompte J, Berlow EL, Brown JH, Fortelius M, et al. (2012): Approaching a state shift in Earth’s biosphere. Nature. 2012;486(7401): 52–58. doi: 10.1038/nature11018 22678279

[6] Wu J. Key concepts and research topics in landscape ecology revisited: 30 years after the Allerton Park workshop. Landscape Ecology. 2013;28(1): 1–11.

[7] Haddad NM, Brudvig LA, Clobert J, Davies KF, Gonzalez A, Holt RD et al. Habitat fragmentation and its lasting impact on Earth’s ecosystems. Science advances 2015;1(2): e1500052. doi: 10.1126/sciadv.1500052 26601154 PMC4643828

[8] MacArthur RH, Wilson EO. The Theory of island biogeography. Princeton Univ. Press. 1967.

[9] Wintle BA, Kujala H, Whitehead A, Cameron A, Veloz S, Kukkala A, et al. Global synthesis of conservation studies reveals the importance of small habitat patches for biodiversity. PNAS. 2019;116(3): 909–914. doi: 10.1073/pnas.1813051115 30530660 PMC6338828

[10] Fahrig L, Arroyo-Rodriguez V, Bennett JR, Boucher-Lalonde V, Cazetta E, Currie DJ, et al. Is habitat fragmentation bad for biodiversity? Biological Conservation. 2019;230: 179–186.

[11] Lawrence A, Hoffmann S, Beierkuhnlein C. Topographic diversity as an indicator for resilience of terrestrial protected areas against climate change. Global Ecology and Conservation. 2020; e01445.

[12] Dudley N, Shadie P, Stolton S. Guidelines for applying protected area management categories including IUCN WCPA best practice guidance on recognising protected areas and assigning management categories and governance types. 2008. Available from https://portals.iucn.org/library/node/30018.

[13] Gray CL, Hill S, Newbold T, Hudson L, Börger L, Contu S. Local biodiversity is higher inside than outside terrestrial protected areas worldwide. Nature Communications 2016;7(12306). doi: 10.1038/ncomms12306 27465407 PMC4974472

[14] UNEP-WCMC (United Nations Environment Programme-World Conservation Monitoring Centre), IUCN (International Union for Conservation of Nature), NGS (National Geographic Society). Protected Planet Report 2018. Cambridge, UK; Gland, Switzerland; Washington, D.C., USA. 2018.

[15] European Commission (EU). Natura 2000; 2019. Available from https://ec.europa.eu/environment/nature/natura2000/index_en.htm.

[16] Popescu VD, Rozylowicz L, Niculae IM, Cucu AL, Hartel T. Species, Habitats, Society: An Evaluation of research supporting EU’s Natura 2000 network. PLoS One. 2014; 9(11): e113648. doi: 10.1371/journal.pone.0113648 25415188 PMC4240592

[17] CEC (Council of the European Communities) Council Directive 92/43/EEC of 21 May 1992 on the conservation of natural habitats and of wild fauna and flora. Official Journal of the European Union. 1992;206: 7–50.

[18] CBD (Convention on Biological Diversity). Strategic Plan for Biodiversity 2011–2020 –COP 10, decision X/2. Convention on Biological Diversity. 2010. Available from http://www.cbd.int/decision/cop/?id=12268, checked on 09/18/2019.

[19] Maes J, Paracchini ML, Zulian G, Dunbar MB, Alkemade R. Synergies and trade-offs between ecosystem service supply, biodiversity, and habitat conservation status in Europe. Biological Conservation. 2012;155: 1–12.

[20] Maiorano L, Falcucci A, Boitani L. Size-dependent resistance of protected areas to land-use change. Proceedings: Biological sciences. 2008;275(1640): 1297–1304. doi: 10.1098/rspb.2007.1756 18319213 PMC2602674

[21] Joppa LN, Pfaff A. High and far: biases in the location of protected areas. PloS ONE. 2009;4(e8273). doi: 10.1371/journal.pone.0008273 20011603 PMC2788247

[22] Hoffmann S, Beierkuhnlein C, Field R, Provenzale A, Chiarucci A. Uniqueness of Protected Areas for Conservation Strategies in the European Union. Scientific, 2018;8(6445). doi: 10.1038/s41598-018-24390-3 29691423 PMC5915414

[23] Kenig-Witkowska MM. Natura 2000-the European Union mechanism for nature conservation: some legal issues. Journal of comparative Urban Law and Policy. 2017;2(1): 198–214.

[24] Laurance WF, Useche DC, Rendeiro J, Kalka M, Bradshaw CJA, Sloan SP, et al. Averting biodiversity collapse in tropical forest protected areas. Nature. 2012;489(7415): 290–294. doi: 10.1038/nature11318 22832582

[25] Clerici N, Bodini A, Eva H, Grégoire JM, Dulieu D, Paolini C. Increased isolation of two Biosphere Reserves and surrounding protected areas (WAP ecological complex, West Africa). Journal for Nature Conservation. 2007;15(1): 26–40.

[26] Watling JI, Nowakowski AJ, Donnelly MA, Orrock JL. Meta-analysis reveals the importance of matrix composition for animals in fragmented habitat. Global Ecology and Biogeography. 2011;20(2): 209–217.

[27] Reider IJ, Donnelly MA, Watling JI. The influence of matrix quality on species richness in remnant forest. Landscape Ecology 2018;33: 1147.

[28] Ricketts TH. The matrix matters: effective isolation in fragmented landscapes. The American naturalist. 2001;158(1): 87–99. doi: 10.1086/320863 18707317

[29] Doherty TS, Driscoll DA. Coupling movement and landscape ecology for animal conservation in production landscapes. Proceedings of the Royal Society B. 2017;(285): e20172272. 10.1098/rspb.2017.2272 PMC5784197 29298935

[30] Brooks TM, Pimm SL, Oyugi JO. Time Lag between Deforestation and Bird Extinction in Tropical Forest Fragments. Conservation Biology. 1999;13(5): 1140–1150.

[31] Diserens TA, Borowik T, Nowak S, Szewczyk M, Niedzwiecka N, Myslajek RW. Deficiencies in Natura 2000 for protecting recovering large carnivores: A spotlight on the wolf Canis lupus in Poland. PLoS One. 2017; 12(9): e0184144. doi: 10.1371/journal.pone.0184144 28873090 PMC5584752

[32] Geldmann J, Joppa LN, Burgess ND. Mapping change in human pressure globally on land and within protected areas. Conservation biology: the journal of the Society for Conservation Biology. 2014;28(6): 1604–1616. doi: 10.1111/cobi.12332 25052712

[33] Jones KR, Venter O, Fuller RA, Allan JR, Maxwell SL, Negret PJ, et al. One-third of global protected land is under intense human pressure. Science. 2018;360(6390): 788–791. doi: 10.1126/science.aap9565 29773750

[34] Perelló LFC, Guadagnin DL, Maltchilk L, Santos JE. Ecological, legal, and methodological principles for planning buffer zones. Natureza & Conservação, 2012;10(1): 3–11.

[35] Selva N, Kreft S, Kati V, Schluck M, Jonsson BG, Mihok B, et al. Roadless and low-traffic areas as conservation targets in europe. Environmental Management. 2011; 48: 865–877. doi: 10.1007/s00267-011-9751-z 21947368 PMC3189408

[36] Orlikowska EH, Roberge JM, Blicharska M, Mikusinski G. Gaps in ecological research on the world’s largest internationally coordinated network of protected areas: A review of Natura 2000. Biological Conservation. 2016; 200: 216–227.

[37] Fahrig L. Effects of habitat fragmentation on biodiversity. Annual Review of Ecology, Evolution, and Systematics. 2003; 34: 487–515.

[38] Fletcher RJ Jr, Didham RK, Banks-Leite C, Barlow J, Ewers RM, Rosindell J, et al. Is habitat fragmentation good for biodiversity?. Biological conservation. 2018;226: 9–15.

[39] Saura S. The Habitat Amount Hypothesis implies negative effects of habitat fragmentation on species richness. Journal of Biogeography. 2020;48: 11–22.

[40] Fahrig L. What the habitat amount hypothesis does and does not predict: A reply to Saura. Journal of Biogeography. 2021;48: 1530–1535.

[41] Hanski I. Habitat fragmentation and species richness. Journal of Biogeography. 2015;42: 989–993.

[42] Fahrig L. Rethinking patch size and isolation effects: The habitat amount hypothesis. Journal of Biogeography. 2013;40: 1649–1663.

[43] Saura S. The habitat amount hypothesis predicts that fragmentation poses a threat to biodiversity: A reply to Fahrig. Journal of Biogeography. 2021;48: 1536–1540.

[44] Haddad NM, Gonzalez A, Brudvig LA, Burt MA, Levey DJ, Damschen EI. Experimental evidence does not support the Habitat Amount Hypothesis. Ecography. 2017;40: 48–55.

[45] Fahrig L. Ecological responses to habitat fragmentation per se. Annual Reviw of Ecology, Evolution and Systematics. 2017; 48: 1–23.

[46] EEA (European Environment Agency). Landscape fragmentation pressure from urban and transport infrastructure expansion. 2018. Available from https://www.eea.europa.eu/data-and-maps/indicators/mobility-and-urbanisation-pressure-on-ecosystems/assessment.

[47] EEA (European Environment Agency). Natura 2000 data—the European network of protected sites. [Data set]. 2018. Available from https://www.eea.europa.eu/data-and-maps/data/natura-9#tab-additional-information.

[48] Jaeger JAG. Landscape division, splitting index, and effective mesh size: new measures of landscape fragmentation. Landscape Ecology. 2000;15(2): 115–130.

[49] Roch L, Jaeger JAG. Monitoring an ecosystem at risk: What is the degree of grassland fragmentation in the Canadian Prairies? Environmental Monitoring and Assessment. 2014; 186(4): 2505–2534. doi: 10.1007/s10661-013-3557-9 24389841

[50] Jaeger JAG, Bertiller R, Schwick C. Degree of Landscape Fragmentation in Switzerland: Quantitative analysis 1885–2002 and implications for traffic planning and regional planning. Neuchâtel: Federal Statistical Office (FSO). 2007.

[51] Jaeger JAG, Soukup T, Madriñán LF, Schwick C, Kienast F. Landscape fragmentation in Europe. Joint EEA-FOEN report. European Environment Agency (EEA), editor. Luxembourg: Publications Office of the European Union. 2011.

[52] Moser B, Jaeger JAG, Tappeiner U, Tasser E, Eiselt B. Modification of the effective mesh size for measuring landscape fragmentation to solve the boundary problem. Landscape Ecology. 2007; 22: 447–459.

[53] EEA (European Environment Agency). Landscape fragmentation data calculated for the landscape fragmentation report by Jaeger et al. (2011). 2014. [Data set].

[54] ESTAT—Eurostat, GISCO, European Commission. Nomenclature of Territorial Units for Statistics (NUTS) 2016—Statistical Units. [Data set]. 2018. Available from https://ec.europa.eu/eurostat/web/gisco/geodata/reference-data/administrative-units-statistical-units.

[55] United Nations Environment Programme-World Conservation Monitoring Centre (UNEP–WCMC). The World Database on Protected Areas. 2019. Available from http://www.protectedplanet.net.

[56] Alexandre B, Crouzeilles R, Grelle CEV. How Can We Estimate Buffer Zones of Protected Areas? A Proposal Using Biological Data. Natureza & Conservacao. 2010; 8(2): 165–170.

[57] Holland JD, Bert DG, Fahrig L. Determining the spatial scale of species’ response to habitat. BioScience. 2004; 54(3): 227–233.

[58] Cai M, Pettenella D. Protecting biodiversity outside protected areas: Can agricultural landscapes contribute to bird conservation on Natura 2000 in Italy? Journal of environmental engineering and landscape management. 2013; 21(1): 1–11.

[59] Hermoso V, Moran-Ordonez A, Brotons L. Assessing the role of Natura 2000 at maintaining dynamic landscapes in Europe over the last two decades: Implications for conservation. Lanscape Ecology. 2018; 33 1447–1460.

[60] R Core Team. A language and environment for statistical computing. R Foundation for Statistical Computing. 2017. Available from https://www.R-project.org/.

[61] Votsi N, Mazaris A, Kallimanis A, Zomeni M, Vogiatzakis I, Sgardelis S, et al. Road effects on habitat richness of the Greek Natura 2000 network. Nature Conservation. 2012;1: 53–71.

[62] Friedrichs M, Hermoso V, Bremerich V, Langhans SD. Evaluation of habitat protection under the European Natura 2000 conservation network–The example for Germany. PLoS One. 2018; 13(12): e0208264. doi: 10.1371/journal.pone.0208264 30566452 PMC6300216

[63] Grodziska-Jurczak M, Strzelecka M, Kamal S, Gutowsk J. Effectiveness of Nature Conservation—A Case of Natura 2000 Sites in Poland. In: Sladonja Barbara, editor. Protected Area Management. InTech. 2012. Available from: https://www.intechopen.com/books/protected-area-management/effectiveness-of-nature-conservation-a-case-of-natura-2000-sites-in-poland

[64] Beunen R, van Assche K, Duineveld M. Performing failure in conservation policy: The implementation of European Union directives in the Netherlands. Land Use Policy 2013;31: 280–288.

[65] Davis M, Naumann S, McFarland K, Graf A, Evans D. Literature review: the ecological effectiveness of the Natura 2000 Network. ETC/BD report to the EEA. 2014;30.

[66] OpenStreetMap. 2021. Available from https://www.openstreetmap.org/.

[67] Rubio-Salcedo M, Martínez I, Carreño F, Escudero A. Poor effectiveness of the Natura 2000 network protecting Mediterranean lichen species. Journal for Nature Conservation. 2013;21(1): 1–9.

[68] Trochet A, Schmeller D. Effectiveness of the Natura 2000 network to cover threatened species. Nature Conservation. 2013;4(1): 35–53.

[69] Lisón F, Sánchez-Fernández D. Low effectiveness of the Natura 2000 network in preventing land-use change in bat hotspots. Biodiversity and Conservation. 2017;26(8): 1989–2006.

[70] Communication department of the European Commission. Countries. 2020 Available from: https://europa.eu/european-union/about-eu/countries_en (12/2020).

[71] Trombulak SC, Frissell CA. Review of ecological effects of roads on terrestrial and aquatic communities. Conservation Biology. 2000;14: 18–30.

