# Supplementary figures and images for: Landscape fragmentation of the Natura 2000 network and its surrounding areas
Source: PLoS One. 2021 Oct 21;16(10):e0258615. doi: 10.1371/journal.pone.0258615 (PMC8530314; doi:10.1371/journal.pone.0258615)

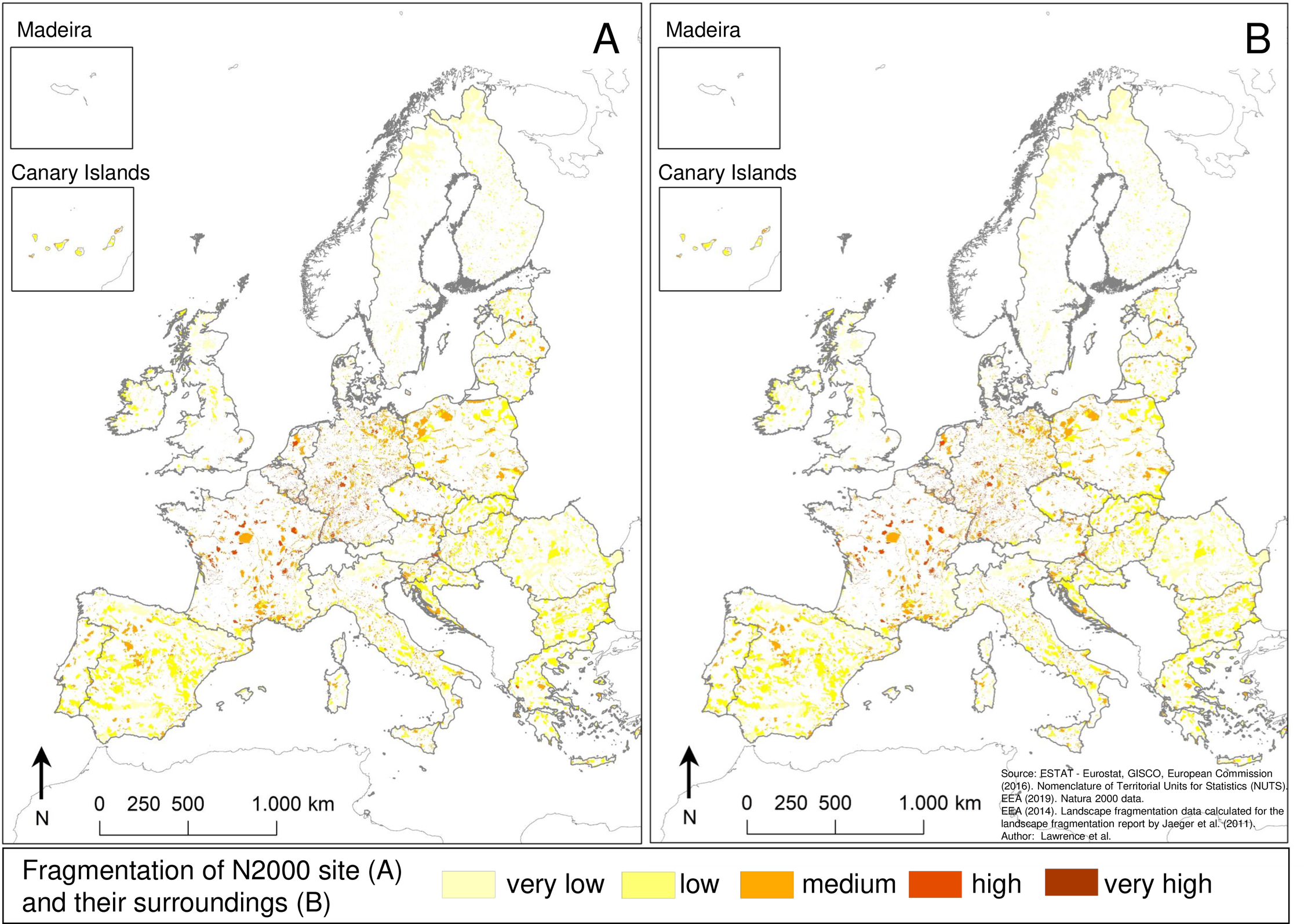

Supplement: S1 Fig — Fragmentation within N2k sites (A) and in their surroundings (B). We calculated seffwithin (A) and seffsurrounding (B) for each N2k site. The coloration of N2k sites represents one of five fragmentation categories (Table 3). Map generated in ArcGIS 10.6.1 (http://www.esri.com/software/arcgis/arcgis-for-desktop). (TIF) [file pone.0258615.s001.tif]
